# Analysis and Simulation of Polymer Injectivity Test in a High Temperature High Salinity Carbonate Reservoir

**DOI:** 10.3390/polym13111765

**Published:** 2021-05-27

**Authors:** Mohamed Adel Alzaabi, Juan Manuel Leon, Arne Skauge, Shehadeh Masalmeh

**Affiliations:** 1 Deptartment of Chemistry, University of Bergen (UiB), 5007 Bergen, Norway; arne.skauge@uib.no; 2 Abu Dhabi National Oil Company (ADNOC), Abu Dhabi P.O. Box 898, United Arab Emirates; jleonhinestrosa@adnoc.ae (J.M.L.); smasalmeh@adnoc.ae (S.M.); 3 Energy Research Norway, 5007 Bergen, Norway

**Keywords:** chemical EOR, polymer flooding, polymer injectivity, in situ rheology, polymer simulation

## Abstract

Polymer flooding has gained much interest within the oil industry in the past few decades as one of the most successful chemical enhanced oil recovery (CEOR) methods. The injectivity of polymer solutions in porous media is a key factor in polymer flooding projects. The main challenge that faces prediction of polymer injectivity in field applications is the inherent non-Newtonian behavior of polymer solutions. Polymer in situ rheology in porous media may exhibit complex behavior that encompasses shear thickening at high flow rates in addition to the typical shear thinning at low rates. This shear-dependent behavior is usually measured in lab core flood experiments. However, data from field applications are usually limited to the well bottom-hole pressure (BHP) as the sole source of information. In this paper, we analyze BHP data from field polymer injectivity test conducted in a Middle Eastern heterogeneous carbonate reservoir characterized by high-temperature and high-salinity (HTHS) conditions. The analysis involved incorporating available data to build a single-well model to simulate the injectivity test. Several generic sensitivities were tested to investigate the impact of stepwise variation in injection flow rate and polymer concentration. Polymer injection was reflected in a non-linear increase in pressure with injection, and longer transient behavior toward steady state. The results differ from water injection which have linear pressure response to rate variation, and quick stabilization of pressure after rate change. The best match of the polymer injection was obtained with complex rheology, that means the combined shear thickening at high rate near the well and moving through apparent Newtonian and shear thinning at low rate.

## 1. Introduction

In polymer flooding chemical EOR methods, viscous polymer solutions are injected in oil reservoirs to improve sweep efficiency of water flooding by reducing mobility ratio between injected water and reservoir oil. The increased viscosity of the polymer solution comes with the expense of reducing well injectivity. Generally, it is essential for any reservoir flooding process to estimate well injectivity with sufficient accuracy and under realistic injection conditions based on reservoir properties. Underestimating injectivity may result in injecting below required target rate and thus affecting project economics and objectives. On the other hand, overestimating injectivity can cause unwanted fracturing of the well. Polymer injectivity can be challenging to estimate due to interference with other factors occurring in an injection well located in the oil zone of the reservoir. Pre-tests with water injection may improve the calibration prior to the polymer test.

Unlike water, polymer solutions often observe a shear-dependent viscosity that generally follow a shear thinning pattern in bulk viscosity measurements, i.e., viscosity decreases with increasing shear rate. Nevertheless, numerous experiments and studies have shown that partially hydrolyzed polyacrylamide (HPAM), which is by far the most widely applied polymer in CEOR projects, exhibits a behavior characterized by apparent shear thickening above a critical shear rate in porous media. Many researchers have studied the factors that could increase or decrease apparent shear thickening in HPAM in lab-scale flow in porous medium experiments. A detailed theoretical analysis of contraction / expansion occurring in porous medium was first reported by Chauveteau (1981) [1], and the early literature on this topic was summarized by Sorbie K.S. (1991) [2]. Later pore network models have elaborated the Chauveteau observations more extensively as in Zamani et al., (2015) [3] and (2017) [4]. Skauge, A. et al., (2018) [5] have summarized the effects of polymer concentration, polymer molecular weight, solution salinity, degree of hydrolysis, pressure, temperature, and porous media properties, on the onset and magnitude of apparent shear thickening in HPAM solutions. Beside in situ rheology, inaccessible pore volume (IPV) and polymer retention due to adsorption and/or mechanical entrapment of polymer molecules may provide extra resistance to the flow and hence impair polymer injectivity. The impact of these factors is usually measured by the residual resistance factor (RRF), which is a measure of permeability reduction due to polymer retention in invaded reservoir zones.

Most lab experiments are conducted using nearly homogeneous samples under controlled conditions. The data obtained from these experiments are therefore abundant and can precisely describe polymer in situ behavior. In contrast, data from field applications are rather limited and the only source of data is usually the injection BHP. Jacobsen, J. et al., (2020) [6] have demonstrated that it is viable to use pressure data to measure in situ polymer rheology in radial flow systems. Their work was based on modeling and history matching radial polymer flow experiments conducted by Skauge et al., (2015) [7] and (2016) [8] on Bentheimer sandstone disc using HPAM. A Detailed analysis of the in situ rheology method for radial flow has been presented by Jacobsen et al., (2020) [9]. Moreover, Alzaabi, M. et al., (2020) [10] attempted to upscale these results to a generic field scale model in which various in situ rheology curves were tested at different injection rates. They concluded that injection BHP can be utilized to evaluate polymer in situ rheology given that rate variation was implemented in injectivity test procedure. They also investigated the impact of vertical heterogeneity and RRF and found that a BHP signal robustly correlates to the specific in situ rheology.

Injectivity tests on single wells are typically conducted prior to multi-well or full-field implementation of polymer flooding to establish the injectivity index of target reservoirs and assure that polymer can be pumped at targeted rates without issues. During injectivity tests, polymer solution is injected at multiple rates and/or concentrations to observe BHP response and gain information about reservoir effective permeability and skin zone radius. The majority of polymer flooding projects worldwide were conducted in sandstone reservoirs rather than carbonate reservoirs, mainly in low-temperature low-salinity formations. Sheng et al., (2015) [11] found that among 733 polymer flooding projects they surveyed, only one seventh was implemented in carbonate reservoirs. Manrique et al., (2017) [12] stated that no polymer flooding projects in carbonate formations were documented after 1990. This is mainly due to lack of polymers for high temperature reservoirs, but also due to the complex geology, heterogeneity, and relatively low permeability of carbonate formations compared to sandstones. Moreover, thermal stability and salinity tolerance of polymer solutions are among major limitations for applications in HTHS reservoirs [13]. Most of the proposed screening criteria for polymer flooding applications in the literature suggest a temperature below 100 °C and formation water salinity below 100,000 ppm for a successful implementation [11]. Recent studies have shown that stability of HPAM could be improved by introducing 2-Acrylamido-2-Methyl Propane Sulfonate (AMPS) as a copolymer [13,14,15,16,17,18]. They reported excellent thermal stability and salinity tolerance of SAV10, a high-AMPS-content acrylamide polymer manufactured by SNF, at temperatures between 120 and 140 °C and salinities up to 244,000 ppm.

This paper is utilizing a systematic approach by analysis gradually build up to match the results of the field trial. The simulation used a commercial reservoir simulator IMEX by Computer Modelling Group ltd (CMG) to predict SAV10 polymer in situ rheology by history matching BHP data of a field injectivity test performed in a Middle Eastern heterogeneous carbonate reservoir. The test was the first of its kind to be performed ever in a HTHS carbonate formation. For more details about the injection scheme and the field trial, see Rachapudi et al., 2020 [19] and Hinestrosa and Masalmeh, 2021 [20].

## 2. Field Polymer Injectivity Test Summary

The single-well polymer injectivity test was performed as part of a larger project to implement full-field polymer flooding in a giant carbonate reservoir in the Middle East that is characterized by harsh temperature and salinity conditions up to 120 °C and 250,000 ppm, respectively [19,20]. The objective of the test was to evaluate polymer injectivity at target rates and concentrations in order to obtain information that would help optimizing the design of later stages in the project.

The reservoir is a Lower Cretaceous carbonate formation characterized by relatively high heterogeneity and stratigraphic cyclicity [21]. Reservoir’s average thickness ranges from 45 to 90 m. The major challenge that drove toward considering polymer flooding option in this reservoir is that it is divided into two main layers that have significant permeability contrast [22]. By implementing polymer injection in high permeability layer, vertical sweep efficiency can be improved by reducing crossflow between the two layers and thus preventing water channeling in high permeability layer and bypassing of oil in the low permeability layer.

According to Rachapudi et al., 2020 [19], a single water injection well was completed in the targeted high permeability zone with a perforation interval of 20 m. Water injection baseline of 13 months was then established prior to polymer injection with rates ranging between 80 and 1,300 m^3^/day. During water injection, multi-rate production logging tool (PLT) logs were conducted to assess vertical injection distribution. Two acid stimulation jobs were also conducted to improve injectivity index. Subsequently, polymer injection phase started and spanned over 4.5 months period. Polymer injection was conducted on several sequences with variable polymer concentrations and injection rates. Moreover, injected polymer went through pre-shearing through dedicated shearing device prior to injection. Degree of pre-shearing ranged between 10% and 50%.

Two pressure fall off (PFO) tests were conducted during polymer injection phase to evaluate skin build-up and in situ effective viscosity. PFOs interpretation through two-layer radial composite models showed increasing skin impairment with time and estimated in situ effective polymer viscosity of 3 mPa.s. Chase water injection continued after concluding polymer injection for about nine months.

## 3. Simulation Approach

A single-well radial model built in CMG IMEX commercial simulator was used to simulate and analyze the polymer injectivity test. The Single-well radial model was adapted and modified from initial interpretation by Hinestrosa and Masalmeh (2021) [20] to improve BHP and vertical distribution history match. The objective of the simulation approach in this study is to exclusively prove the concept of polymer in situ rheology prediction through injection BHP data. Therefore, the workflow adopted for the objective of this study was exclusively as follows:

- Establish reliable model inputs by history matching water injection baseline BHP.
- Test BHP sensitivity to rate and/or concentration stepping with generic in situ rheology curves.
- Investigate the impact of RRF dependence on permeability and sensitivity to different permeability-RRF correlations.
- Use in situ rheology and RRF as key parameters to history match polymer injection and chase water BHP.
- Compare obtained polymer behavior to lab data.

### 3.1. Model Description

**Figure A1:**
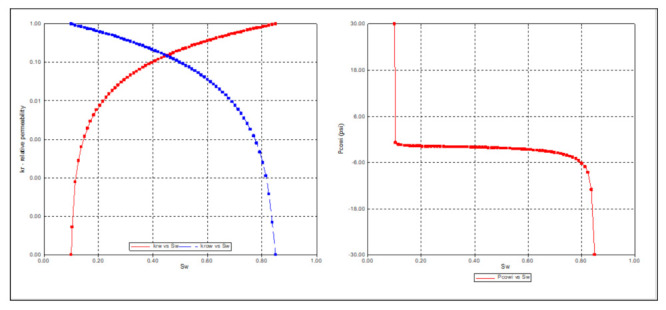
Oil-water relative permeability and capillary pressure curves for permeability above 100 mD (Modified and adapted from [21]).

**Figure A2:**
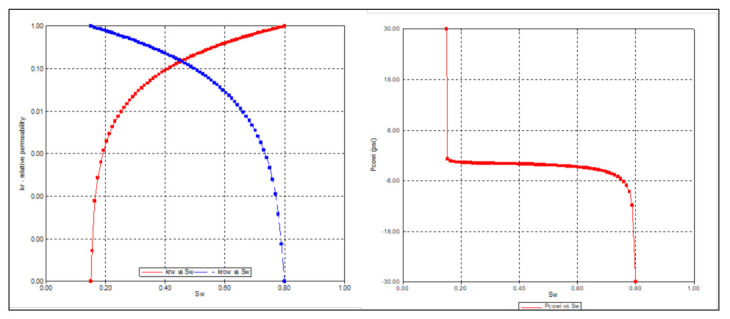
Oil-water relative permeability and capillary pressure curves for permeability below 100 mD (Modified and adapted from [21]).

Choosing radial coordinates over Cartesian is recommended in polymer injectivity modeling as it prevents velocity smearing in the near-well bore region and hence allow more accurate recognition of in situ rheology curves input. The model has 20 grids in the radial direction and 89 layers. A dummy producer was placed in the outermost grid for material balance purposes. Number of layers was based on provided up-scaled geological and petrophysical data. Fine gridding was applied in the near wellbore region to capture high Darcy velocities in wellbore vicinity. A summary of model parameters is shown in Table 1 below. Static inputs and assumptions for petrophysical, PVT, and rock-fluid data are shown in Table A1 and Figure A1 and Figure A2.

### 3.2. Polymer Properties

The SAV 10 polymer is a synthetic polymer based on HPAM, but with very high content of 2-Acrylamido-2-Methyl Propane Sulfonate (AMPS). The polymer is produced by SNF, Andrézieux-Bouthéon, France. The molecular weight of the samples used is about 8 million g/mol, but the product has been produced in a range of molecular weights [13,14,15].

### 3.3. Water Injection Baseline History Match Approach

Considering radial Darcy equation for flow in porous media:

(1) $$\Delta P=\frac{Q\;\mu }{2\;\pi \;k\;h}\ln\left(\frac{r}{r_{w}}\right)+s$$

where ΔP is the pressure drop between an injector with radius of $r_{w}$ and a point at r distance in a reservoir with thickness of h, Q is injection rate, μ is injected fluid viscosity, and s is the skin factor; we could assume that that the main two parameters of concern to match water injection baseline are permeability and skin factor due to their relatively significant uncertainty. Data used for permeability distribution input were obtained from core data of off-set wells and corrected through indirect conversion of porosity and saturation logs. Due to this uncertainty and due to reservoir inherent heterogeneity, it is a common practice to apply permeability multipliers to the original input in order to match BHP response. PLT logs conducted during water injection baseline were utilized to justify proposed permeability multipliers. Six PLT sets were available for analysis of which three were conducted during water injection before acid stimulation jobs, one after acid stimulation, and two during polymer injection.

### 3.4. Impact of Rate and Concentration Stepping

Several generic cases with different rate and concentration stepping scenarios were tested over the actual period of polymer injection phase. The objective was to investigate the sensitivity to different rate and concentration stepping at Newtonian and non-Newtonian conditions.

The following generic scenarios were considered:

- Constant rate and constant concentration
- Concentration steps at constant rate
- Rate steps with:
  ○ Constant concentration
  ○ Increasing concentration
  ○ Decreasing concentration

Rate and concentrations assumed in the above scenarios are shown in Table 2 below.

For Newtonian rheology cases, water was injected at 0.43 mPa.s viscosity, which is the viscosity of seawater used in the actual test. The rheology curves corresponding to non-Newtonian behavior were created using an extended version of Carreau model [2,23] that is used to fit complex polymer rheology including shear-thinning and shear-thickening:

(2) $$\mu_{app}=\mu_{\infty }+\left[\left(\mu_{o}-\mu_{\infty }\right)*{\left[1+{\left(\lambda_{1}u\right)}^{2}\right]}^{\frac{n_{1}-1}{2}}\right]+\left[\mu_{max}*\left(1-e^{-{\left[\lambda_{2}u\right]}^{n_{2}-1}}\right)\right]$$

where $\mu_{app}$ is polymer apparent viscosity, $\mu_{o}$ and $\mu_{\infty }$ are limiting Newtonian viscosities at high and low shear limits, respectively, λ and n are empirical polymer constants, *u* is the superficial velocity of the polymer in porous media and $\mu_{max}$ is the shear-thickening plateau viscosity.

**Figure 1:**
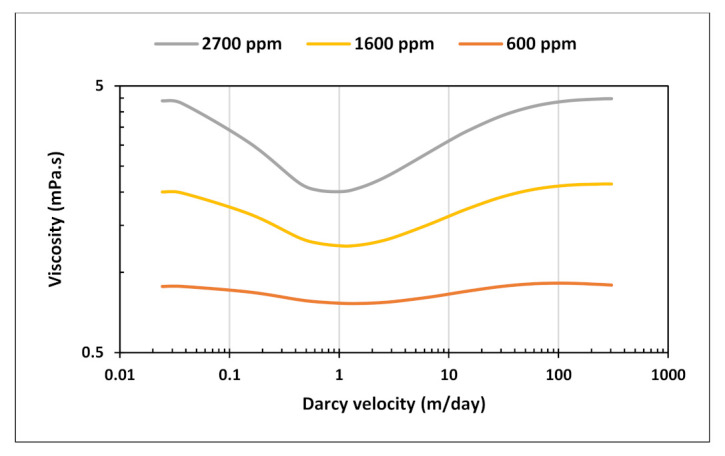
In situ rheology curves used in the rate and concentration stepping sensitivity.

Three curves were created to cover polymer concentrations applied in the test (Figure 1). The generated curves exhibit a combined shear effect that decreases with decreasing concentration toward a near-Newtonian behavior. Velocity range of the curves is between 0.03 and 300 m/day. The outcomes of simulations with rate and/or concentration steps will demonstrate the sensitivity of BHP when encountering various segments of complex rheology depending on calculated Darcy velocity values at each rate. Parameters of rheology model equation used to generate the curves is in Table 3 below.

### 3.5. Impact of Residual Resistance Factor (RRF)

Permeability reduction due to polymer adsorption in porous media is measured by RRF, which is the ratio of water mobility before to after polymer flood.

(3) $$RRF=\frac{\lambda_{w,init}}{\lambda_{w,p}}=\frac{k_{w,init}}{k_{w,p}}$$

**Figure 2:**
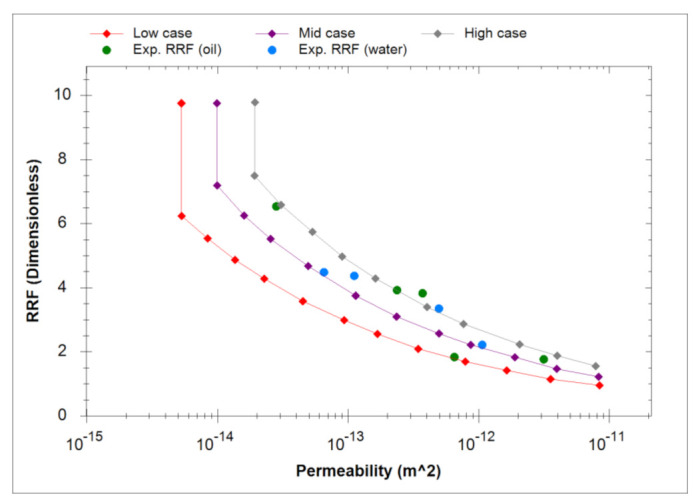
Proposed low, mid, and high RRF correlations based on lab data (Modified from [20]).

In general, the level of polymer adsorption increases in tighter formations due to increase in the fraction of pore-volume inaccessible to larger polymer molecules. The relationship governing RRF dependence on permeability is thus considered as a major tuning parameter for history matching polymer flooding. Several RRF-permeability correlations were tested based on lab-measured RRF data of SAV-10 polymer as proposed by Leon and Masalmeh, 2021 [20]. (Figure 2). Besides permeability-dependent RRF, average RRF values are often used in modelling polymer flooding to simplify history matching process. This method is usually more suitable for homogenous reservoirs; however, it may be applicable for heterogeneous reservoirs considering average formation capacity. Therefore, BHP response to weighted average RRF values of proposed correlations corresponding to the layers’ permeability and thickness was also investigated.

### 3.6. Polymer Injection History Matching Approach

**Figure 3:**
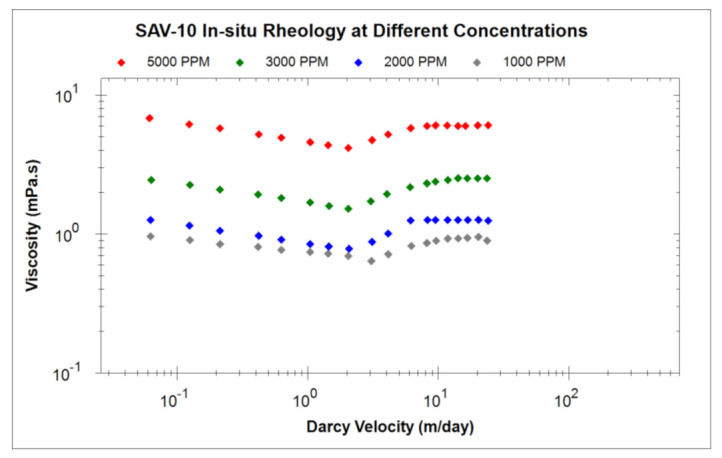
In situ viscosity measurements of SAV-10 at different concentration at 120 °C (Modified from [20]).

**Figure 4:**
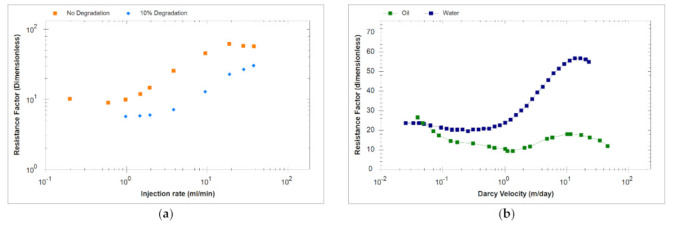
(**a**) Impact of degradation from pre-shearing and (**b**) Oil presence, on the onset of SAV-10 apparent shear thickening in porous media (Modified from [20]).

The polymer injection phase was matched using information gained from generic sensitivity studies and integration of available lab and field data. In situ measurements that incorporate the impact of shear rate on SAV10 viscosity at different concentrations are shown in Figure 3. Experimental results show apparent shear thickening at high shear rates for flow in porous media, proving the inherent complex rheology behavior of SAV10. Besides, the effects of pre-shearing and oil presence were also investigated as shown in Figure 4a,b. It is evident from these results that both pre-shearing and presence of oil can reduce the degree of shear thickening and delay its onset to higher shear rates [16,20].

**Figure 5:**
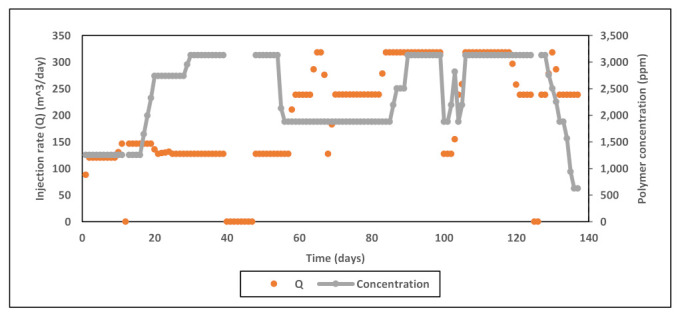
Data of injection rates and polymer concentrations used in the injectivity test.

**Figure 6:**
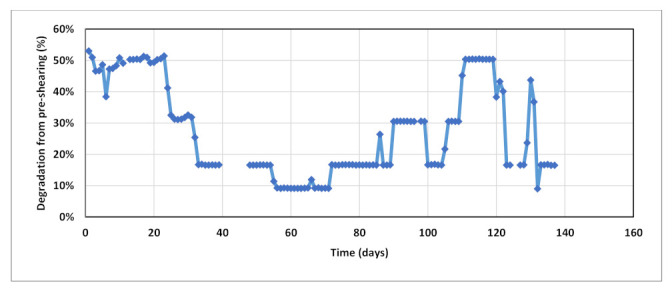
Percentage of degradation from pre-shearing applied on polymer solution prior to injection.

**Figure 7:**
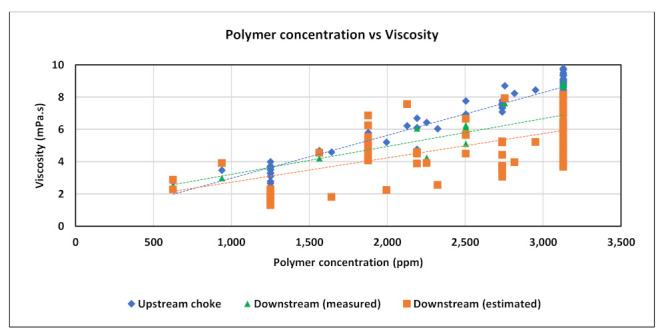
Polymer viscosity measurements across the shearing device choke and estimated downstream viscosities.

Field data used in this study include daily records of injection rates, injected concentrations, viscosity measurements across shearing device, degradation % from pre-shearing, and BHP. Four main injection rate steps were performed during the test at 127, 238, 318, and 238 m^3^/day as shown in Figure 5 below. Concentration stepping was implemented only during first sequence at 127 m^3^/day. The second and third sequences had nearly constant concentrations, and the last sequence was dedicated for concentration tapering toward the end of the test. The test program also included pre-shearing the polymer solution prior to injection (Figure 6). Viscosity measurements were conducted upstream the shearing device choke for the whole test period. Downstream viscosity measurements were conducted when the well was operating at vacuum i.e., zero wellhead pressure. For periods when wellhead pressure is not zero, downstream viscosity was estimated from shearing device calibration correlation. Figure 7 shows both measured and estimated viscosity measurements upstream and downstream the shearing device. It is evident that measured downstream viscosities deviate slightly and are larger than the viscosities estimated from correlation. In addition, one can observe that the impact of pre-shearing is more pronounced at high concentrations. In order to account for the impact of pre-shearing, the inputs for polymer concentrations in the model were reduced to mimic degradation % and therefore recognize viscosity reduction due to degradation.

## 4. Results and Discussion

### 4.1. Water Injection Baseline History Matching

**Figure 8:**
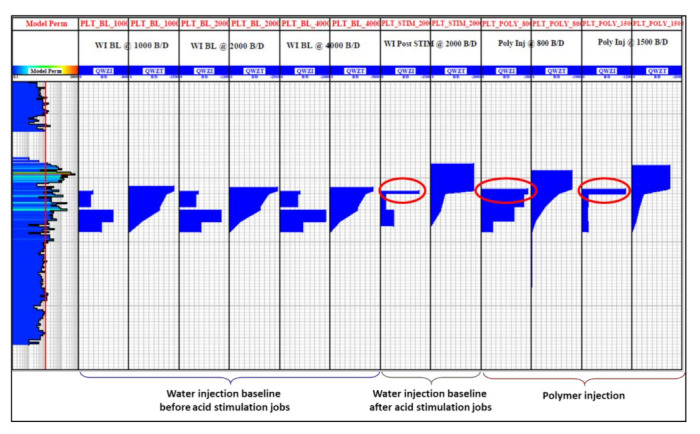
Production logging tool (PLT) logs conducted during injectivity test (Modified from [20]).

**Figure 9:**
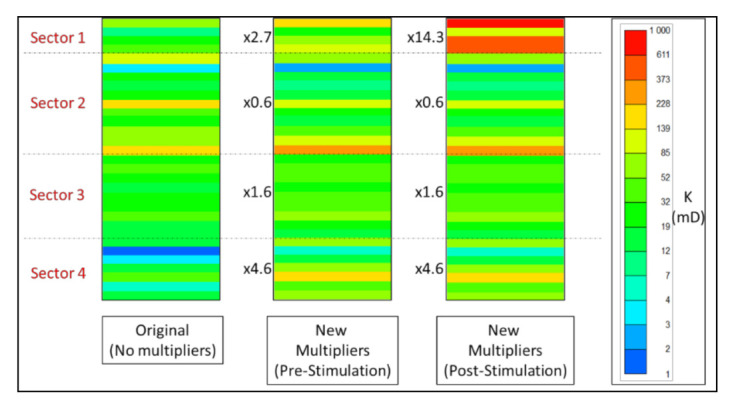
Permeability multipliers applied to match PLT logs pre- and post- acid stimulation jobs.

**Figure 10:**
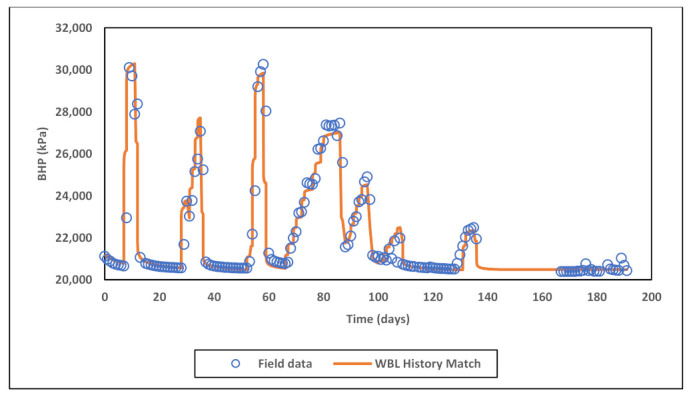
History match of water injection baseline BHP.

It is evident from PLT results (Figure 8), that the uppermost sector of perforated section received significantly more injected fluid after acid stimulation jobs, this can indicate severe permeability alteration possibly as a result of an induced fracture or wormhole activation. Therefore, a dynamic permeability technique was applied to alter upper sector permeability multiplier by using simulation restart method. The objective was to match PLT logs of water injection baseline period in order to correct vertical permeability distribution. Figure 9 below shows the applied permeability multipliers and comparison to original data. Sectors within perforated zone were defined based on the results of PLT. For skin factor tuning, best matches were obtained with the following assumptions: (1) +3.5 skin before first acid stimulation, (2) +2.0 skin after first acid stimulation, and (3) −0.45 skin with permeability alteration in the upper sector after the second acid stimulation. The BHP history match for water injection baseline is shown in Figure 10.

### 4.2. Sensitivity to Rate and Concentration Stepping

**Figure 11:**
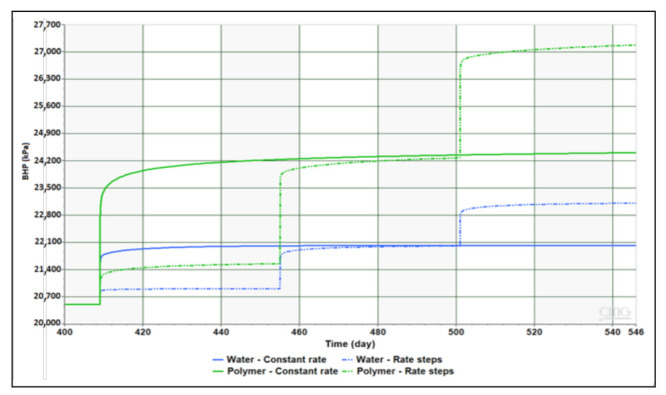
Impact of rate stepping on Newtonian and non-Newtonian injection.

**Figure 12:**
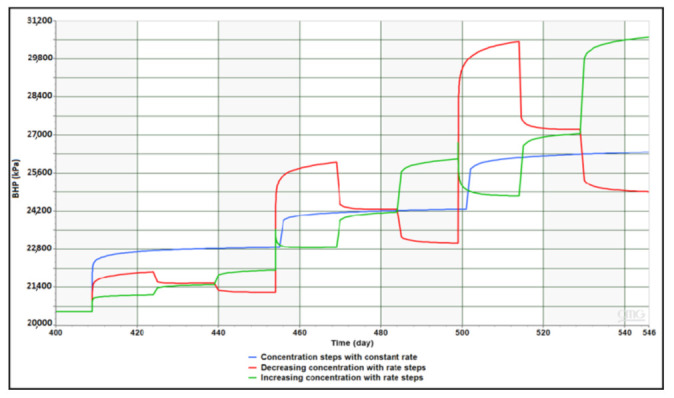
Impact of concentration stepping with different patterns on polymer injection.

Results obtained from generic simulations of BHP sensitivity to rate (Q) and concentration (Cp) stepping are shown in Figure 11 and Figure 12, respectively. Findings had confirmed that non-Newtonian injection takes a significantly longer time to stabilize compared to Newtonian. This behavior is observed for both constant rate injection as well as in rates stepping case. Besides, shear thickening behavior in near-wellbore region is detectable through the gradual increase in BHP at every rate step indicating increase in viscosity with increasing Darcy velocity. The near-Newtonian behavior of BHP at the lowest rate step is reflecting the Newtonian plateau that exists between shear thickening and shear thinning segments in the rheology curve of applied concentrations. Concentration stepping has shown no impact of the concentration change direction whether it is increasing or decreasing as pressure increases and drops at the same magnitude. However, the transition of BHP response between concentration steps demonstrates more gradual trend when compared to the one between rate steps.

### 4.3. Sensitivity to Residual Resistance Factor (RRF)

**Figure 13:**
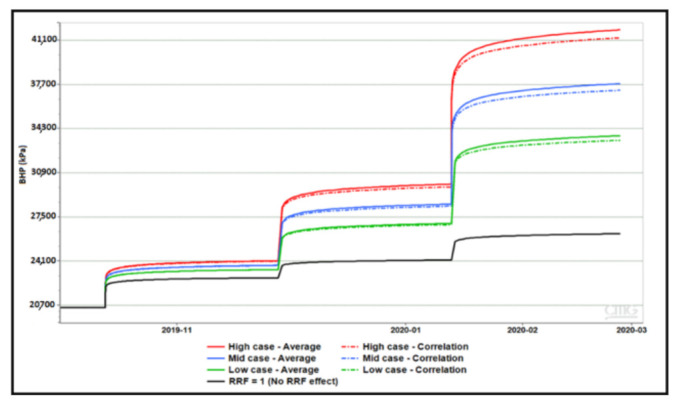
Impact of RRF correlations and averages on concentration stepping.

**Figure 14:**
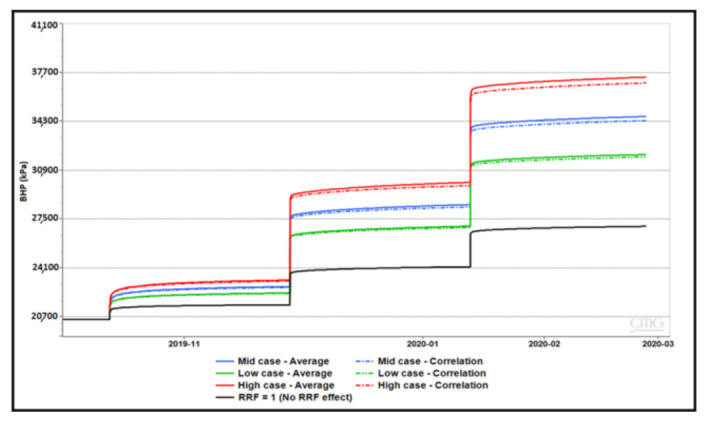
Impact of RRF correlation and averages on rate stepping.

Several permeability dependent RRF correlations were proposed based on provided lab data. For each correlation, weighted average RRF was calculated using weighted formation capacity based on layer thickness and permeability. Table 4 below show the three correlations and their respective weighted average RRF. Results of BHP sensitivity to each case with concentration stepping and rate stepping are shown in Figure 13 and Figure 14, respectively. Simulations have demonstrated that correlation and weighted average RRF’s result in the similar pressure response. Besides, the observed effects of concentration stepping and rate stepping are not affected by applied RRF.

### 4.4. Analysis of Field Bottom-Hole Pressure Data

It is evident from previous research findings [2,4], that non-Newtonian rheology behavior results in non-linear transient BHP response with longer stabilization time compared to Newtonian behavior. Injection of Newtonian fluid (like water) yields a linear response and significantly shorter buildup of BHP toward stabilization. One can detect these behaviors from plots of BHP versus injection rates and BHP versus time.

**Figure 15:**
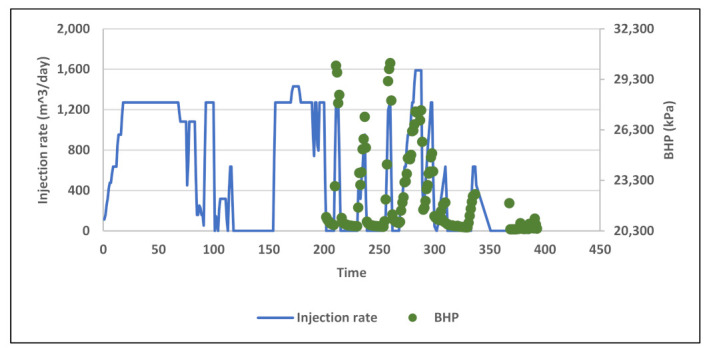
Field data of injection rates and BHP during water injection baseline.

**Figure 16:**
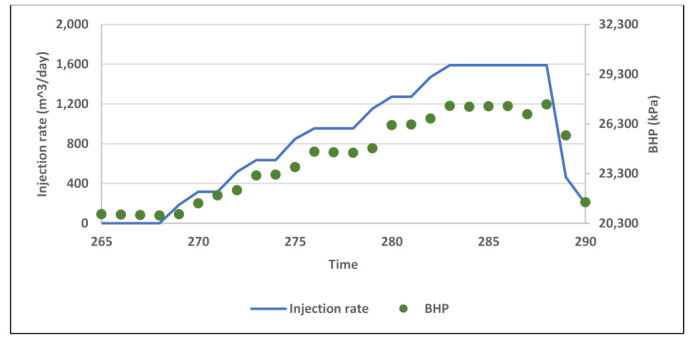
Segment of water injection baseline used for BHP analysis with rate steps.

**Figure 17:**
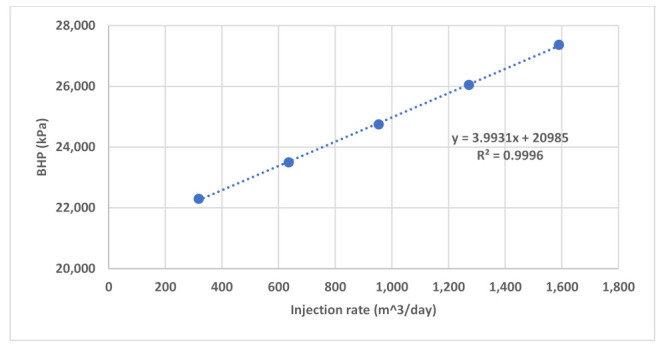
Plot of BHP versus injection rate for part of water injection baseline.

Field data of BHP during water injection baseline and polymer injection were analyzed to investigate BHP response to Newtonian and non-Newtonian flow. Although water injection took place over 13 months, pressure data is available only for the second half of that period (Figure 15). Besides that, the data see extreme rate fluctuations and very short, interrupted injection periods. There is also the acid stimulation jobs impact which significantly affects pressure response. Therefore, the only analyzable pressure data found was in period after first acid stimulation where water injection took place for about 20 days with five 300 m^3^/day rate steps up to 1600 m^3^/day (Figure 16). The plot of BHP versus injection rate shows a linear correlation which reflects Newtonian behavior (Figure 17).

**Figure 18:**
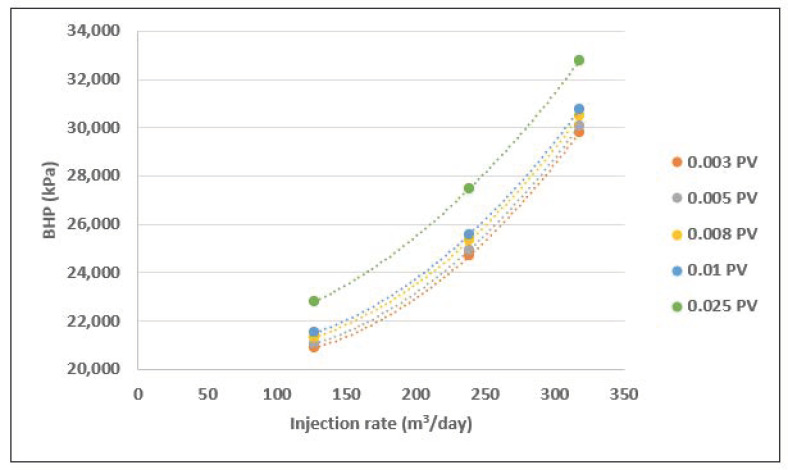
BHP versus injection rate for polymer injection at several pore-volumes.

Oppositely, the analysis of BHP response during polymer injection shown a signal of active apparent shear-thickening behavior. This is demonstrated through plots of BHP vs. injection rate at several injected pore-volumes (PV’s) (Figure 18). The increasing slope is a predicted signature of shear thickening as viscosity increases with Darcy velocity.

**Figure 19:**
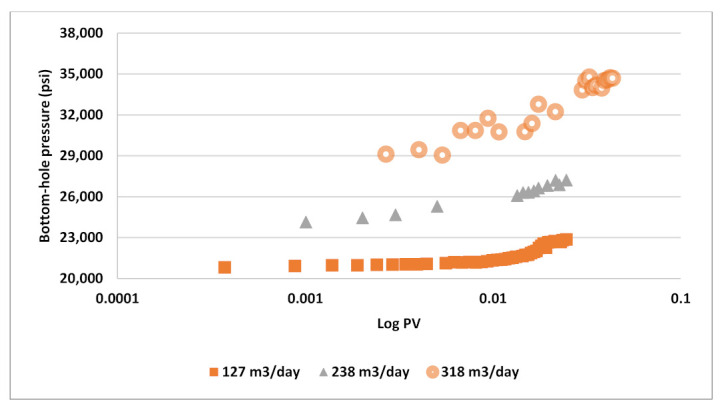
BHP versus log pore-volume at different injection rates.

The BHP response versus time was also analyzed for each rate step as shown in Figure 19. The pressure profiles versus log PV exhibit sharp increases especially for higher rates (238 and 318 m^3^/day) which is considered as a signal of shear thickening behavior in the near well bore region. At the rate of 127 m^3^/day, one can notice that BHP increase in larger slope at higher concentrations. This evidence supports the assumption of the rheology leaning toward near-Newtonian behavior with decreasing concentration. In contrast to these observations, shear thinning would demonstrate decreasing slope in BHP vs. rate plots and gradual increase in BHP with time as demonstrated by Alzaabi et al., 2020 [4].

### 4.5. History Matching Polymer Injection and Chase Water

**Figure 20:**
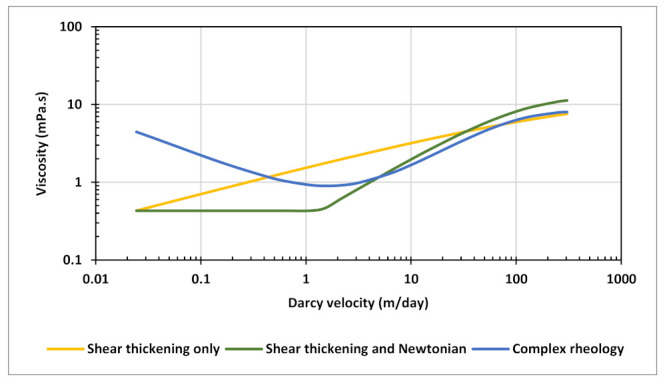
Shear thickening rheology scenarios tested for polymer injection history matching.

Following above results and findings, three scenarios that include only shear thickening behavior at high velocities were assumed for polymer injection history matching (Figure 20). The rheology curves were created using extended Carreau equation with the same average viscosity of 3 mPa.s over the selected velocity range. These assumed scenarios are inclusive of all possible shear thickening behaviors in the near-well bore region. In CMG IMEX simulator, complex combined polymer rheology is defined through velocity tables. Each velocity table corresponds to a specific Darcy velocity value, under which user can input values for polymer concentration and corresponding viscosity. The simulator calculates water-polymer mixture velocity for each grid block and performs a two-dimensional interpolation to calculate relative polymer viscosity based on relative concentration. Therefore, the defined curves are all corresponding to a single concentration value, the maximum concentration in this case, and the simulator performs interpolations for lower concentrations. This implies that rheology curves for lower concentrations are essentially parallel shifted curves with the same slope as the defined curve.

**Figure 21:**
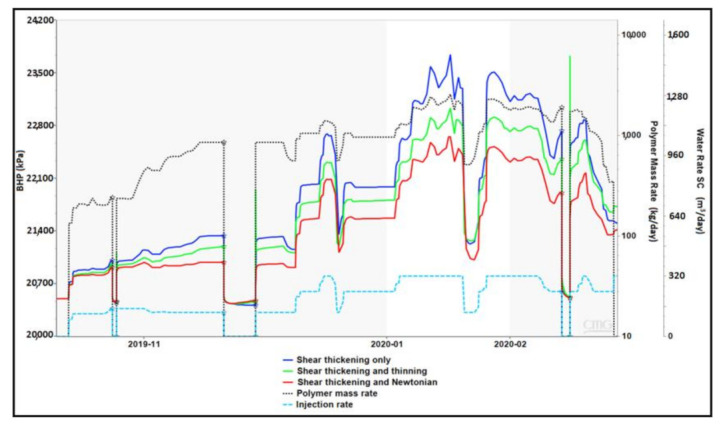
BHP response of the shear thickening cases using actual field rates and concentrations.

The model was used to simulate actual polymer injection period with field injection rates and polymer concentrations using the three generic shear thickening cases. Results of simulated BHP are shown in Figure 21 along with injection rate and injected polymer mass rate. It is evident that the shear thickening only scenario results in the highest pressure build up since it involves ever-increasing viscosity in near-wellbore region toward the injector. The combined effect scenario where shear thinning takes place at low velocities exhibits a slightly lower pressure response. This may indicate that the most acting velocity range is actually between 0.3 to 30 m/day where the viscosity of shear thickening only case is larger. The pressure response is overall reflecting the trend of polymer concentration represented by polymer mass rate. However, due to different slopes of applied in situ rheology curves, the pressure contrasts with concentration and/or rate variations are more pronounced for lower slopes. Moreover, the combined rheology with shear thinning at low velocities shows better representation for gradual changes in rate and concentration. It does however exhibit spiking pressure response at the points of injection resumption after shut-in periods which might be a simulator artifact.

**Figure 22:**
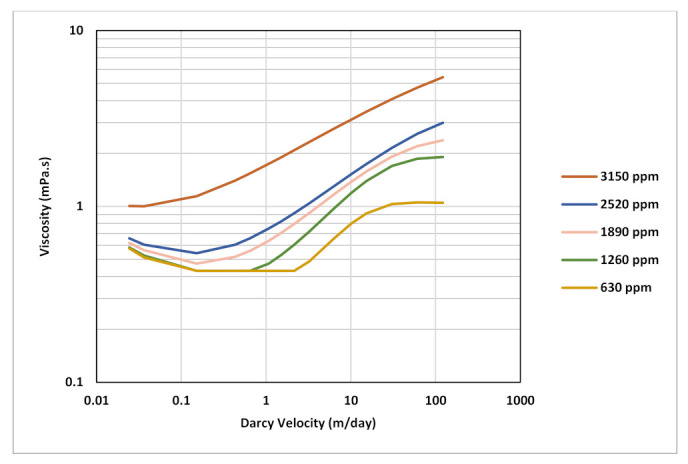
Rheology curves used in history matching polymer injection.

Considering above analyses and findings, best match for polymer injection phase was obtained using a combined rheology effect with five-curve input representing five different concentration levels (Figure 22). The curves were generated using extended Carreau equation with the parameters shown in Table 5. The applied rheology exhibits a delay of shear thickening onset and larger slope as concentration decreases which reflects the impact of degradation from pre-shearing. Moreover, low concentrations have more pronounced Newtonian plateaus at high and low velocity endpoints. Maximum viscosities at highest velocities of the five curves ranged from 5 mPa.s to 1 mPa.s. A shear thinning component was also included and found essential for history matching with all curves having identical shear thinning parameters.

**Figure 23:**
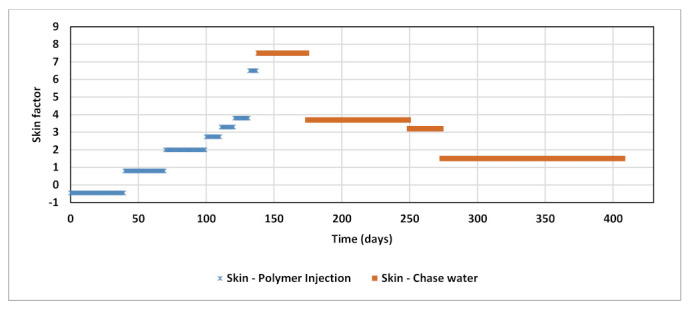
Skin factor applied along polymer injection and chase water in history match.

**Figure 24:**
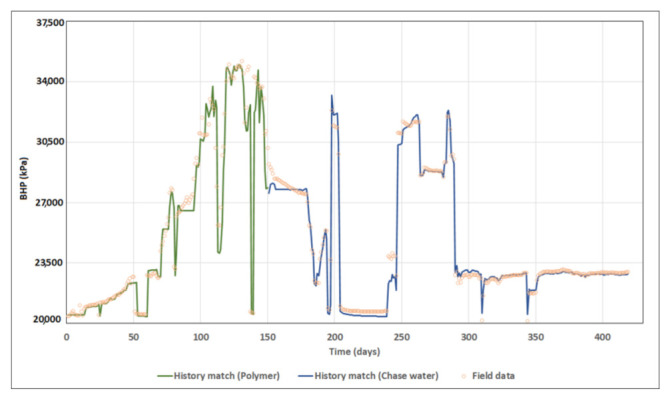
History matching BHP of polymer injection and chase water.

Both polymer injection and chase water matchings were achievable with an average RRF value of “4.1”. which is the weighted average RRF value of the proposed high case correlation. A dynamic skin factor was essentially applied to mimic the skin impairment increase. Skin was thus updated monthly starting from −0.45 up to +7.5. The skin impairment was assumed as a temporary impact of polymer slug accumulation in the near-wellbore region, therefore better match to the chase water was obtained when skin was gradually reduced to its original prior to polymer injection (Figure 23). The history matched BHP of polymer injection and chase water is illustrated in Figure 24.

## 5. Conclusions

In this paper, a polymer injectivity field test in a high temperature high salinity carbonate reservoir was analyzed through numerical simulation approach utilizing CMG IMEX simulator. The analysis involved investigating the sensitivity of rate and concentration stepping on BHP response as well as impact of permeability correlated RRF. The polymer injection BHP was successfully history matched with a set of in situ rheology curves reflecting the impact of polymer concentration and rheological properties.

The conclusions from this study can be summarized as follows:

- PLT logs of water baseline injection prior to polymer injection can be utilized to match vertical injection distribution across perforated zone. This practice can provide a more accurate permeability inputs especially for cases where significant uncertainty in permeability exists.
- Average RRF values corrected to weighted average formation capacity are sufficient for BHP history matching purposes as they yield similar results as permeability-dependent RRF correlations.
- Date from field downhole measurements of BHP versus injection rates can be utilized to detect in situ fluid rheology. Newtonian water injection showed linear trend while polymer injection showed a non-linear trend with increasing slope reflecting shear thickening behavior.
- The degree of degradation due to pre-shearing can be represented in the model by reducing injected concentration by the same percentage and applying multiple rheology realizations to account for degradation impact.
- The non-Newtonian behavior in the near-wellbore region can be distinguished from the Newtonian behavior by the characteristics of longer transient pressure build up due to the velocity-dependent viscosity.

## Figures and Tables

**Table 1:** Single-well model parameters.

Grid Type	Radial
**Well type**	Vertical
**Grid dimensions**	20 × 1 × 89
**Innermost grid size**	0.08 m
**Outermost grid size**	343 m
**Total radius**	914 m
**Layer thickness**	0.38–3.66 m
**Total thickness**	90 m
**Perforated section**	Layers 23 to 55 (20 m)

**Table 2:** Rates and concentrations used for stepping generic scenarios.

Scenario	Value(s)
**Constant rate**	480 m^3^/day
**Rate steps**	160, 480, and 795 m^3^/day
**Constant concentration**	1,600 ppm
**Concentration steps**	600, 1,600, and 2,700 ppm

**Table 3:** Parameters used to generate in situ rheology curves for sensitivity tests.

	2700 ppm	1600 ppm	600 ppm
*u*	0.02 to 300 m/day		
*µ_max_*	10	4.5	1.35
*n_2_*	1.5		
*λ_2_*	1.00 E + 04		
*µ_∞_*	0.43		
*µ_0_*	10	4.5	2
*n_1_*	0.2	0.5	0.8
*λ_1_*	1.00 E + 06		

**Table 4:** Correlations proposed to fit RRF lab data with their respective weighted average.

Scenario	RRF Correlation	K_min_ (×10^−15^ m^2^)	Weighted Average RRF
**Low**	$RRF=9.6\times {\left(k\right)}^{-0.251}$	5	4.171
**Mid**	$RRF=13.0\times {\left(k\right)}^{-0.255}$	10	3.322
**High**	$RRF=16.4\times {\left(k\right)}^{-0.255}$	20	2.506

**Table 5:** Extended Carreau equation parameters used to create matching rheology curves.

	3150 ppm	2520 ppm	1890 ppm	1260 ppm	630 ppm
*u*	0.02 to 120 m/day				
*µ_max_*	17	8	5.5	4	2
*n_2_*	1.36	1.52	1.6	1.75	2.2
*λ_2_*	1.2 E + 03	2.0 E + 03	4.0 E + 03	6.0 E + 03	1.0 E + 04
*µ_∞_*	0.43				
*µ_0_*	2				
*n_1_*	0.5				
*λ_1_*	1.00 E + 07				

## Appendix A

**Table A1:** PVT data used in the model.

Parameter	Value
Reservoir temperature	120 °C
Bubble point pressure	14,755 kPa
Oil density	815.18 Kg/m^3^
Oil viscosity	0.32 mPa.s
Water density	1174.79 Kg/m^3^
